# Integrated traditional Chinese medicine alleviates sciatica while regulating gene expression in peripheral blood

**DOI:** 10.1186/s13018-021-02280-1

**Published:** 2021-02-11

**Authors:** Yi Wang, Guogang Dai, Yan Xu, Ling Jiang, Zhibin Fu, Jiao Xia, Guogang Tian, Wanli Du

**Affiliations:** 1 Cervicodynia/Omalgia/Lumbago/Sciatica Department 2, Sichuan Provincial Orthopedics Hospital, No. 132 West First Section First Ring Road, Wuhou District, Chengdu, Sichuan Province China; 2 Experiment Teaching Center for Preclinical Medicine, Chengdu Medical College, No. 783, Xindu Avenue, Xindu District, Chengdu, Sichuan Province China; 3 College Hospital, Sichuan Agricultural University, Chengdu Campus, No. 211 Huiming Road, Wenjiang District, Chengdu, Sichuan Province China; 4 Department of Lower Extremities, Sport Hospital Affiliated to Chengdu Sport Institute, No. 2, Tiyuan Road, Wuhou District, Chengdu, Sichuan Province China

**Keywords:** Sciatica, Peripheral blood, Gene expression, Traditional Chinese medicine

## Abstract

**Background:**

Although integrated traditional Chinese medicine (TCM) has long been indicated to be effective in the treatment of sciatica and is widely used in the management of this condition, the mechanism by which integrated TCM alleviates sciatica has not yet been fully defined, and the effect of integrated TCM on gene expression in the peripheral blood of patients with sciatica is still unknown. We performed this study to investigate the effect of integrated TCM on peripheral blood gene expression in patients with sciatica and to explore new clues for studying the mechanism of integrated TCM in alleviating sciatica.

**Methods:**

We used a microarray to identify differentially expressed genes (DEGs) in the peripheral blood of patients with sciatica and healthy controls (DEGs-baseline), bioinformatic analysis to reveal the characteristics of DEGs-baseline, and the key genes that contribute to the gene dysregulation. A microarray was also used to identify DEGs in the peripheral blood of patients with sciatica after integrated TCM treatment compared with those at baseline, and the expression levels of DEGs were validated by qRT-PCR.

**Results:**

We identified 153 DEGs-baseline, which included 131 upregulated genes and 22 downregulated genes. Bioinformatic analysis revealed that most of the DEGs-baseline were related to immunity and the inflammatory response and that TLR4, MMP9, MPO, CAMP, RETN, TLR5, and IL1RN were key genes involved in the dysregulation of genes in the peripheral blood of patients with sciatica. The expression levels of TLR5, IL1RN, SLC8A1, RBM20, GPER1, IL27, SOCS1, and GRTP1-AS1 were decreased in the peripheral blood of patients after integrated TCM treatment compared with that at baseline, which was accompanied by relief of pain.

**Conclusion:**

Integrated TCM treatment relieved pain while regulating the gene expression of TLR5, IL1RN, SLC8A1, RBM20, GPER1, IL27, SOCS1, and GRTP1-AS1 in the peripheral blood of patients with sciatica. Our study provides new clues for studying the mechanism of TCM in treating sciatica.

**Supplementary Information:**

The online version contains supplementary material available at 10.1186/s13018-021-02280-1.

## Background

Sciatica is characterized by neuropathic pain in the sciatic nerve, and the lifetime incidence is up to 40% [1]. Lumbar disc herniation is a major cause of sciatica and a major concern in studies about sciatica [2]. It is generally recognized that during lumbar disc herniation, mechanical compression in combination with immunity and inflammation causes sciatica. Many studies have revealed inflammatory transcriptome characteristics of degenerated discs [3–6], and many cytokines related to immunity and inflammation were activated in herniated lumbar discs [7–10]. However, since sciatica involves a focal lesion, gene expression changes in peripheral blood caused by sciatica have rarely been studied.

Many methods have been reported to improve sciatica, and 90% of acute sciatica can be effectively relieved by nonsurgical treatment [11]. Integrated traditional Chinese medicine (TCM) has long been indicated to be effective in the treatment of sciatica and is widely used in the management of this condition [12–14]. However, the mechanism by which integrated TCM relieves sciatica has not yet been fully defined. Therefore, we performed the present study to investigate the gene expression changes after integrated TCM treatment in the peripheral blood of patients with sciatica, aiming to explore new clues for studying the role of traditional Chinese medicine in alleviating sciatica.

## Methods

### Enrollment and integrated TCM treatment

This study was approved by the Ethics Committee of Sichuan Provincial Orthopedics Hospital, and written informed consent was obtained. From April 2018 to December 2019, 25 patients and 25 healthy volunteers were enrolled in this observational study. Patients were enrolled from those who chose to receive nonsurgical treatment with integrated TCM in our hospital. Diagnostic criteria of sciatica was set according to the guidelines of the North American Spine Society [15]: (1) a history of pain in the distribution of sciatic nerve, (2) a positive Lasegue’s sign, and (3) had findings on magnetic resonance imaging of lumbar disc herniation at the L4/5 level or L5/S1 level with compression of the corresponding nerve root. The patient inclusion criteria were as follows: (1) aged between 18 and 60 years, (2) had sciatica secondary to single-level lumbar disc herniation at the L4/5 level or L5/S1 level, and (3) had no medication history within 3 months before the appointment. The exclusion criteria were as follows: (1) concomitant cauda equina syndrome, other neuropathy, other spinal diseases, infection, rheumatism, cardiovascular disease, metabolic disease, hematopoietic diseases, dementia, consciousness disturbance, or mental illness; (2) a history of surgery, congenital disease, tuberculosis, or tumors; (3) pregnancy or lactation; and (4) other medical conditions that might affect gene expression in peripheral blood. Healthy volunteers were recruited as healthy controls. All patients underwent standard integrated TCM treatment for sciatica according to our medical protocol, including electric acupuncture, massage, moxibustion, and Chinese medicine fumigation for waist circumference. The pain intensity score on the visual analogue scale (VAS, ranging from 0 to 10, with higher scores indicating greater pain intensity) was recorded.

### Peripheral blood collection, RNA extraction, and microarray analysis

Peripheral blood was collected and frozen in PAXgene tubes, and then, total RNA was extracted and purified using the PAXgeneTM Blood RNA Kit (Qiagen, Germany). An Agilent SurePrint G3 Microarray (8 × 60 K) was used for the microarray experiment, which was performed by Shanghai Biotech Co. (Shanghai, China). These procedures were described in our previous study [16].

### Differentially expressed genes (DEGs)

Two groups of DEGs were identified: DEGs in the peripheral blood of patients with sciatica and healthy controls (DEGs-baseline) and DEGs in the peripheral blood of patients with sciatica after integrated TCM treatment compared with those at baseline (DEGs-TCM).

Data from the chip scan were log2 normalized for comparative analyses. Unrecognized probes were discarded. We deleted unrecognized probes. The average value of the data from the probes corresponding to the same gene was used for analysis. Genes with an absolute fold change (FC) of ≥ 1.5 were identified as DEGs, and the FC data were filtered by *t* tests (*P* < 0.05). Gene expression data sets are accessible on the Gene Expression Omnibus (GEO) database (http://www.ncbi.nlm.nih.gov/geo) under the number GSE150408 and GSE124272.

### Enrichment analysis of DEGs-baseline

Enrichment analysis was performed with the web-based portal Metascape [17], which included Gene Ontology (GO) analysis of biological processes (BP), cellular components (CC), and molecular functions (MF), and Kyoto Encyclopedia of Genes and Genomes (KEGG) pathway analysis.

### Protein–protein interaction (PPI) network

To identify key genes in the dysregulation of gene expression in the peripheral blood of patients at baseline and in healthy controls, we used the STRING database to construct the PPI network of DEGs-baseline with a combined score > 0.4. Cytoscape software (V3.6.1) was used to visualize the PPI network. Disconnected nodes were excluded, the degree of centrality of the DEGs-baseline was calculated, and a submodule was clustered, as we previously described [16].

### Quantitative real-time quantitative PCR (qRT-PCR) and statistical analysis

qRT-PCR was performed to measure the expression levels of DEGs using the 2^-ΔΔCq^ method. β-Actin was used as the normalization control for mRNA. The design and synthesis of the primers and the qRT-PCR procedure were described previously [16]. The primers are listed in Additional file 1. Significant differences in expression levels were determined using *t* test with a *P* value < 0.05. Statistical analysis was performed and visualized as we previously described [16].

## Results

### Patients and leg-pain scores

We enrolled 25 patients with sciatica aged between 19 and 54 years (mean age 40 years) who were seen in our department for integrated TCM treatment and 25 healthy volunteers aged between 19 and 30 years (mean age 23 years) as healthy controls. All patients underwent standard integrated TCM treatment for sciatica for at least 14 days. The VAS score for pain in patients decreased (*P* < 0.000), from an average of 7.4 ± 0.81 at baseline to 2.1 ± 0.97 14 days after integrated TCM treatment, indicating that all patients achieved clinical remission.

### Microarray analysis

**Fig. 1:**
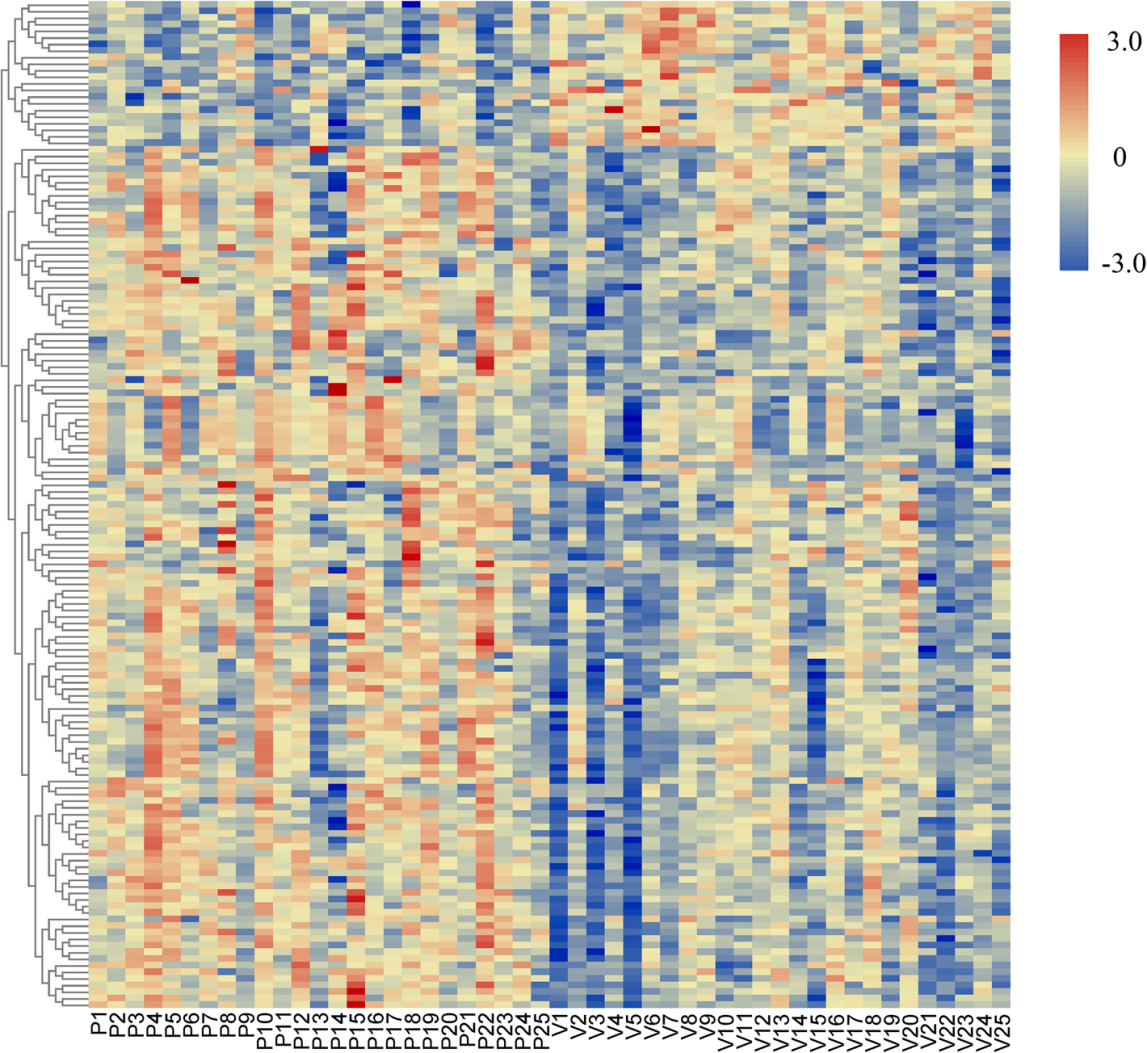
Heatmap of the DEGs in the peripheral blood of patients with sciatica and healthy controls. The expression data were normalized using the z-score for indexes between − 3 and 3. Red, upregulated; blue, downregulated. *P* patient with sciatica, *V* healthy volunteer

Microarray analysis identified 153 DEGs-baseline, which included 131 upregulated genes and 22 downregulated genes (Fig. 1). After integrated TCM treatment, 8 genes were differentially expressed in the peripheral blood of patients compared with those at baseline, and all of these genes were downregulated (Table 1). When we compared the DEGs-baseline and DEGs-TCM, we found that toll-like receptor 5 (TLR5), interleukin-1 receptor antagonist (IL1RN), and solute carrier family 8 member A1 (SLC8A1) were present in both groups (Table 1) and that TLR5, IL1RN, and SLC8A1 were upregulated in sciatica patients at baseline compared with healthy controls and were then downregulated in sciatica patients after integrated TCM treatment compared with baseline.

**Table 1:** Differentially expressed genes. After TCM treatment, 8 genes were differentially expressed in the peripheral blood of patients compared with those at baseline. TLR5, IL1RN, and SLC8A1 were upregulated in sciatica patients at baseline compared with healthy controls and were then downregulated in sciatica patients after TCM treatment compared with baseline.

Gene symbol	Description	Baseline vs. control		TCM vs. Baseline	
		***P*** value	Fold change	***P*** value	Fold change
TLR5	Toll-like receptor 5	0.00	1.6	0.03	0.67
IL1RN	Interleukin 1 receptor antagonist	0.01	1.7	0.03	0.64
SLC8A1	Solute carrier family 8 member A1	0.00	1.5	0.04	0.63
RBM20	RNA binding motif protein 20	> 0.05	-	0.03	0.62
GPER1	G protein-coupled estrogen receptor 1	> 0.05	-	0.00	0.62
IL27	Interleukin 27	> 0.05	-	0.01	0.59
SOCS1	Suppressor of cytokine signalling 1	> 0.05	-	0.02	0.56
GRTP1-AS1	GRTP1 antisense RNA 1	> 0.05	-	0.04	0.48

*TCM* traditional Chinese medicine, *TLR5* toll-like receptor 5, *IL1RN* interleukin 1 receptor antagonist, *SLC8A1* solute carrier family 8 member A1, *RBM20* RNA binding motif protein 20, *GPER1* G protein-coupled estrogen receptor 1, *IL27* interleukin 27, *SOCS1* suppressor of cytokine signalling 1, *GRTP1-AS1* GRTP1 antisense RNA 1

### Enrichment analysis of DEGs-baseline

Enrichment analysis by Metascape revealed that upregulated genes at baseline were enriched in 115 GO BP, 25 GO CC, 4 GO MF, and 6 KEGG pathways (Table 2). Downregulated genes at baseline were enriched in 10 GO BP, 1 GO MF, and 2 KEGG pathways (Table 3). Most of the enriched terms for both upregulated and downregulated DEGs at baseline were related to immunity and the inflammatory response. Enrichment analysis revealed immune and inflammatory response-related gene expression characteristics in the peripheral blood of patients with sciatica.

**Table 2:** Gene Ontology analysis of upregulated DEGs-baseline. The top 15 enriched terms of the upregulated genes in the peripheral blood of the patients with sciatica at baseline compared with the healthy controls according to the *P* value. Log10(P) is the log10-based *P* value.

Term	Category	Description	Log10(P)
GO:0002274	GO BP	Myeloid leukocyte activation	− 19.59
GO:0002366	GO BP	Leukocyte activation involved in immune response	− 17.57
GO:0002263	GO BP	Cell activation involved in immune response	− 17.51
GO:0043312	GO BP	Neutrophil degranulation	− 17.42
GO:0002283	GO BP	Neutrophil activation involved in immune response	− 17.35
GO:0002275	GO BP	Myeloid cell activation involved in immune response	− 17.32
GO:0042119	GO BP	Neutrophil activation	− 17.15
GO:0002446	GO BP	Neutrophil-mediated immunity	− 17.12
GO:0036230	GO BP	Granulocyte activation	− 17.02
GO:0043299	GO BP	Leukocyte degranulation	− 16.44
GO:0002444	GO BP	Myeloid leukocyte-mediated immunity	− 16.10
GO:0045055	GO BP	Regulated exocytosis	− 14.36
GO:0042581	GO CC	Specific granule	− 14.34
GO:0070820	GO CC	Tertiary granule	− 12.84
GO:0035580	GO CC	Specific granule lumen	− 8.99

*GO* gene ontology, *BP* biological processes, *CC* cellular components, *MF* molecular function

**Table 3:** Gene Ontology analysis of downregulated DEGs-baseline. All enriched terms of the downregulated genes in the peripheral blood of the patients with sciatica at baseline compared with the healthy controls according to the *P* value. Log10(P) is the log10-based *P* value.

Category	Term	Description	Log10(P)
KEGG pathway	hsa04612	Antigen processing and presentation	− 4.34
GO MF	GO:0030246	Carbohydrate binding	− 4.07
KEGG pathway	hsa04650	Natural killer cell-mediated cytotoxicity	− 3.63
GO BP	GO:0002708	Positive regulation of lymphocyte-mediated immunity	− 3.92
GO BP	GO:0002705	Positive regulation of leukocyte-mediated immunity	− 3.62
GO BP	GO:0002706	Regulation of lymphocyte-mediated immunity	− 3.46
GO BP	GO:0002703	Regulation of leukocyte-mediated immunity	− 3.08
GO BP	GO:0002699	Positive regulation of immune effector process	− 2.99
GO BP	GO:0002449	Lymphocyte-mediated immunity	− 2.37
GO BP	GO:0045089	Positive regulation of innate immune response	− 2.32
GO BP	GO:0045088	Regulation of innate immune response	− 2.12
GO BP	GO:0002697	Regulation of immune effector process	− 2.08
GO BP	GO:0070997	Neuron death	− 2.40

*GO* gene ontology, *BP* biological processes, *CC* cellular components, *MF* molecular function, *KEGG* Kyoto Encyclopedia of Genes and Genomes

### PPI network

**Fig. 2:**
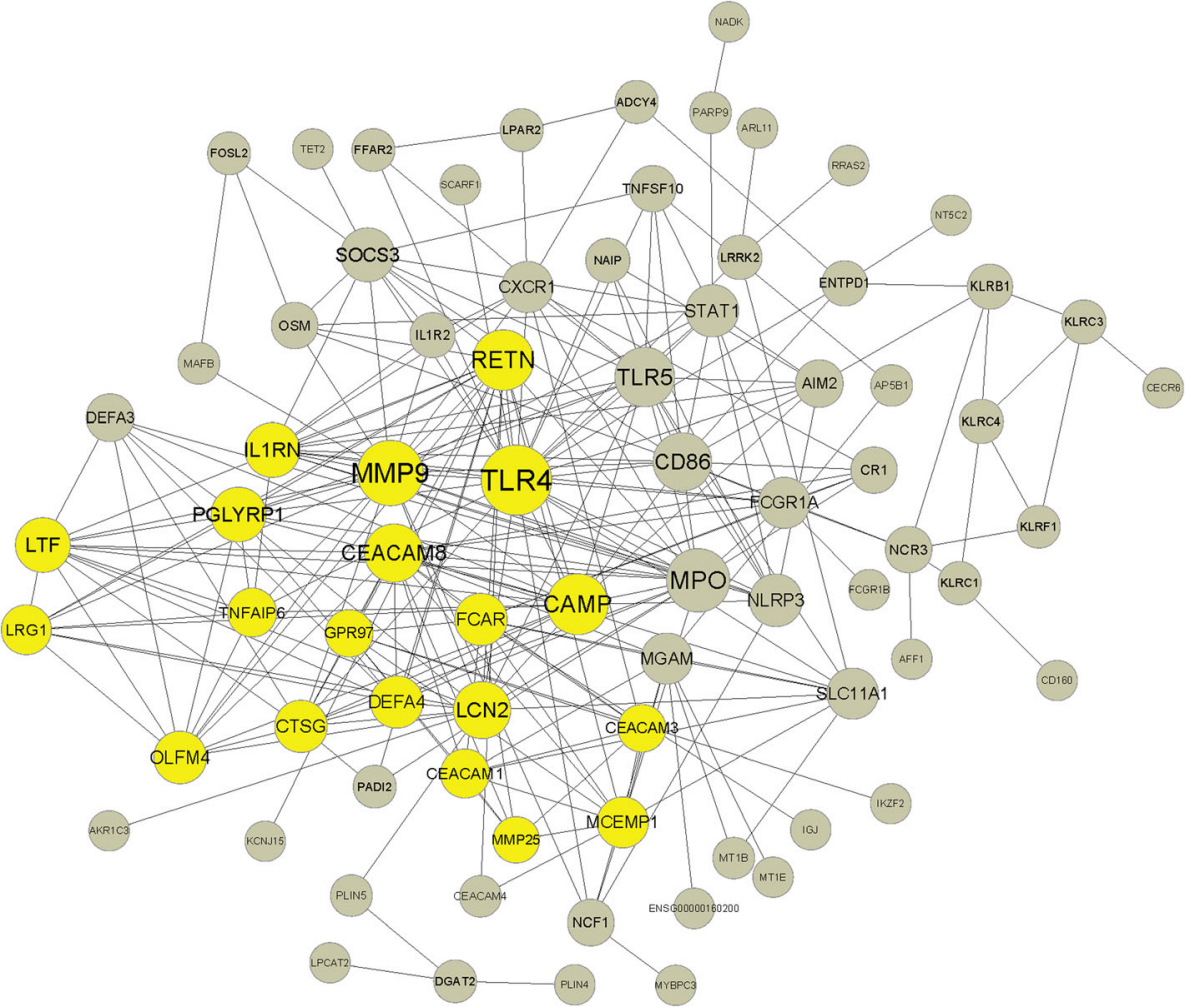
PPI network of DEGs at baseline. Seventy-seven nodes with a combined score > 0.4 and 255 edges were included. The size of the nodes represents the centrality degree. One cluster was identified by MCODE analysis with threshold k-core = 5 and is colored yellow

The PPI network of DEGs-baseline consisted of 77 connected nodes and 255 edges (Fig. 2). To identify key genes in the PPI network, the plug-in CentiScaPe was used to calculate the centrality degree of each node. Genes with a high degree centrality included toll-like receptor (TLR4, degree 26), matrix metallopeptidase 9 (MMP9, degree 22), myeloperoxidase (MPO, degree 20), cathelicidin antimicrobial peptide (CAMP, degree 18), resistin (RETN, degree 18), TLR5 (degree, 17), etc. The top 10 genes with the highest degree centrality are listed in Table 4. MCODE analysis identified 1 cluster in the PPI network with threshold k-core = 5. TLR4, MMP9, CAMP, and IL1RN were enriched in this cluster. The PPI network suggested that TLR4, MMP9, MPO, CAMP, RETN, TLR5, and IL1RN were key genes in the dysregulation of genes in the peripheral blood of patients with sciatica.

**Table 4:** Top 10 genes with the highest centrality degree in the PPI network. Centrality degree was calculated by the CentiScaPe plug-in, and one cluster was identified by MCODE analysis with threshold k-core = 5

Gene	Degree	MCODE_cluster
TLR4	26	Clustered
MMP9	22	Clustered
MPO	20	Unclustered
CAMP	18	Clustered
RETN	18	Clustered
TLR5	17	Unclustered
CEACAM8	16	Clustered
CD86	16	Unclustered
LCN2	15	Clustered
IL1RN	13	Clustered

*TLR4* toll-like receptor 4, *MMP9* matrix metallopeptidase 9, *MPO* myeloperoxidase, *CAMP* cathelicidin antimicrobial peptide, *RETN* resistin, *TLR5* toll-like receptor 5, *CEACAM8* CEA cell adhesion molecule 8, *CD86* CD86 molecule, *LCN2* lipocalin 2, *IL1RN* interleukin 1 receptor antagonist

### Expression of DEGs

We examined the expression levels of the key DEGs-baseline (TLR4, MMP9, MPO, CAMP, RETN, TLR5, and IL1RN) in the peripheral blood of patients at baseline and in the peripheral blood of healthy controls, as well as expression levels of DEGs-TCM in the peripheral blood of patients at baseline and in the peripheral blood of patients after integrated TCM treatment.

**Fig. 3:**
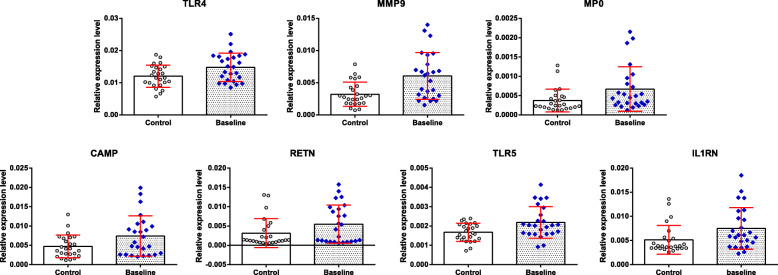
Expression of the key DEGs contributing to dysregulation of gene expression in patients with sciatica. ^*^P < 0.05

**Fig. 4:**
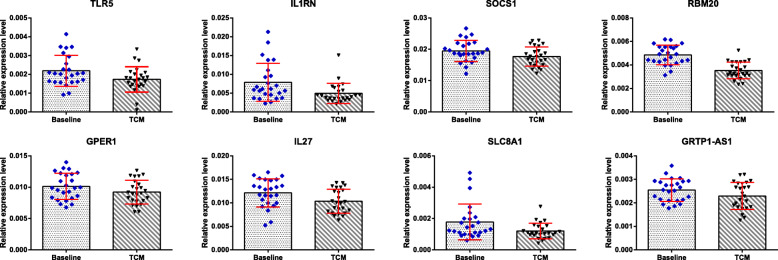
Expression of DEGs of patients with sciatica after integrated TCM treatment compared with those at baseline. ^*^P < 0.05

qRT-PCR showed that the expression levels of TLR4, MMP9, MPO, CAMP, RETN, TLR5, and IL1RN were increased in the peripheral blood of patients at baseline compared with the healthy controls (Fig. 3); the expression levels of TLR5, IL1RN, SLC8A1, RNA binding motif protein 20 (RBM20), G protein-coupled estrogen receptor 1 (GPER1), interleukin 27 (IL27), suppressor of cytokine signalling 1 (SOCS1), and GRTP1 antisense RNA 1 (GRTP1-AS1) were decreased in the peripheral blood of the patients after integrated TCM treatment compared with that at baseline (Fig. 4).

## Discussion

With microarray analysis, we identified 153 DEGs in the peripheral blood of sciatica patients and healthy controls. Bioinformatic analysis revealed that these DEGs were mainly related to immunity and the inflammatory response and that TLR4, MMP9, MPO, CAMP, RETN, TLR5, and IL1R were the most important genes contributing to the dysregulation of these DEGs.

In the PPI network of DEGs-baseline, TLR4 had the highest centrality degree, which was consistent with our previous study [16]. TLR4 is the most studied Toll-like receptor because it plays a broad role in the inflammatory response [18]. It is also involved in innate neuroimmunity and neuropathy and mediates inflammatory and neuropathic pain [19]. An animal model confirmed that the expression of TLR4 in intervertebral discs and activation of TLR4 in intervertebral discs induced an inflammatory response, including the upregulation of tumor necrosis factor α, IL-1β, IL-6, and nitric oxide [20]. The inhibition of TLR4 in discs reduced inflammation and reversed pain-related neuroplasticity, suggesting that TLR4 is a potential target for treating disc-related inflammatory and neuropathic pain [21]. MMP9 is associated with sciatica in both peripheral blood and local discs. MMP9 has long been known to degrade collagen. It is expressed in intervertebral discs, which are full of collagen [22], and the expression levels of MMP9 decrease in free, protruded, and extruded discs [23]. MMP 9 is also involved in the activation and inactivation of inflammation, although the mechanism has not been completely elucidated [24]. Focal MMP9 causes leukocytes to migrate from peripheral blood to tissues by promoting a chemotactic gradient, which has a role in the neuroinflammatory process [25]. MPO is mainly produced by circulating neutrophils and is linked to inflammatory conditions and degenerative neurological disorders. By producing hypochlorous acid-sphingomyelinase, which is released as a source of reactive oxygen species, MPO indirectly mediates inflammatory injury [26]. In stroke patients, MPO expression is increased in both the plasma and the serum [27]. The expression of MPO is increased in the brains of patients with Alzheimer’s or Parkinson’s disease [28, 29], and MPO has been reported to be activated in human multiple sclerosis plaques [30]. The exact mechanism of MPO in these neurological diseases is not completely clear. RETN levels were reported to be associated with inflammatory cytokines, including TNF-α receptor-2, IL-6, ICAM-1, lipoprotein-associated PLA2, and C-reactive protein [31, 32]. RETN also upregulates TNF-α and IL-6 in human peripheral blood mononuclear cells as well as TNF-α and IL-12 in human macrophages [33, 34]. Combining these studies, a potential link between TLR4, MMP9, MPO, CAMP, RETN, and sciatica can be established, but microarray analysis showed that the expression of these genes was not changed after integrated TCM treatment. Integrated TCM treatment does not alleviate sciatica by regulating these genes.

After integrated TCM treatment, the pain of the patients was relieved, and the expression levels of TLR5, IL1RN, SLC8A1, RBM20, GPER1, IL27, SOCS1, and GRTP1-AS1 in peripheral blood were downregulated compared with baseline. We also found that TLR5, IL1RN, and SLC8A1 were upregulated in sciatica patients at baseline compared with healthy controls and downregulated after integrated TCM treatment compared with baseline, indicating that these genes played an important role in the pathogenesis and remission of sciatica. In the future, the expression of these genes can be considered as an objective indicator for evaluating the efficacy of integrated TCM in treating sciatica in the clinical setting.

Animal experiments have shown that the knockout or blockade of TLR5 can reduce pain [35, 36], which is consistent with our finding that TLR5 expression was downregulated in patients with pain relief after integrated TCM treatment. IL1RN polymorphism and genetic variability were reported to be associated with outcomes of intervetebral disc disease [37, 38], but no studies have elaborated the role of IL1RN mRNA expression in sciatica. The roles of SLC8A1, RBM20, GPER1, IL27, SOCS1, and GRTP1-AS1 in sciatica or in relieving sciatica are currently unknown. Future studies should further investigate the role of these genes in sciatica remission.

Our study had limitations. Some other factors, such as miRNAs and lncRNAs, are involved in the process of protein translation from mRNA, and protein expression is not only determined by mRNA. We did not perform western blotting to measure the protein levels of these genes, and the protein levels and mRNA levels of these genes may not be consistent. Changes in gene expression and alleviation of sciatica were simultaneously observed after integrated TCM treatment in the present study; whether integrated TCM treatment relieved sciatica by regulating the expression of these genes still needs further investigation.

## Conclusions

In conclusion, we found that integrated TCM treatment alleviated sciatica while regulating the gene expression of TLR5, IL1RN, SLC8A1, RBM20, GPER1, IL27, SOCS1, and GRTP1-AS1 in the peripheral blood of patients. Our study provides new clues for studying the mechanism of TCM in treating sciatica.

## Supplementary Information

**Additional file 1.** Sequences of primers used for qRT-PCR

## Data Availability

The gene expression datasets generated and analyzed during the current study are available in the GEO database (http://www.ncbi.nlm.nih.gov/geo) under the number GSE150408 and GSE124272. The datasets used and/or analyzed during the current study are available from the corresponding author on reasonable request.

## Abbreviations

TCM: Traditional Chinese medicine
DEGs: Differentially expressed genes
DEGs-baseline: DEGs in the peripheral blood of patients with sciatica and healthy controls
DEGs-TCM: DEGs in the peripheral blood of patients with sciatica after integrated TCM treatment compared with those at baseline
GO: Gene ontology
BP: Biological processes
CC: Cellular components
MF: Molecular functions
KEGG: Kyoto Encyclopedia of Genes and Genomes
PPI: Protein–protein interaction
qRT-PCR: Quantitative real-time quantitative PCR
TLR5: Toll-like receptor 5
IL1RN: Interleukin 1 receptor antagonist
SLC8A1: Solute carrier family 8 member A1
TLR4: Toll-like receptor
MMP9: Matrix metallopeptidase 9
MPO: Myeloperoxidase
CAMP: Cathelicidin antimicrobial peptide
RETN: Resistin
RBM20: RNA-binding motif protein 20
GPER1: G protein-coupled estrogen receptor 1
IL27: Interleukin 27
SOCS1: Suppressor of cytokine signalling 1
GRTP1-AS1: GRTP1 antisense RNA 1
