# Supplementary material for: Integrated traditional Chinese medicine alleviates sciatica while regulating gene expression in peripheral blood
Source: J Orthop Surg Res. 2021 Feb 11;16:130. doi: 10.1186/s13018-021-02280-1 (PMC7877113; doi:10.1186/s13018-021-02280-1)
Supplement: Supplementary file 1 — Additional file 1. Sequences of primers used for qRT-PCR [file 13018_2021_2280_MOESM1_ESM.docx]

**Additional file 1.** Sequences of primers used for qRT-PCR. TLR4: Toll-like receptor 4; MMP9: matrix metallopeptidase 9; MPO: myeloperoxidase; TLR5: Toll-like receptor 5; IL1RN: interleukin 1 receptor antagonist; SLC8A1: solute carrier family 8 member A1. REMB20: RNA binding motif protein 20; GPER1: G protein-coupled estrogen receptor 1; IL27: interleukin 27; SOCS1: suppressor of cytokine signaling 1; GRTP1-AS1: GRTP1 antisense RNA 1; F, forward; R, reverse.

| **Gene** | **Sequence (5' to 3')** |
| --- | --- |
| TLR4 | F: CCTGAGGCATTTAGGCAGCTA |
|  | R: GATAAATCCAGCACCTGCAGTTC |
| MMP9 | F: CACGCACGACGTCTTCCA |
|  | R: AAGCGGTCCTGGCAGAAAT |
| MPO | F: CGGTACCCAGTTCAGGAAGCT |
|  | R: CCCTCGTTCTCCCACCAAA |
| CAMP | F: TCAAGGATTTTTTGCGGAATCT |
|  | R: GCCAGGGTAGGGCACACA |
| RETN | F: AGCCATCAATGAGAGGATCCA |
|  | R: AGGCCAATGCTGCTTATTGC |
| TLR5 | F: TCTGCTAGGACAACGAGGATCA |
|  | R: CCATGAGCACCACTCCTAGGA |
| IL1RN | F: CAGCTGGAGGCAGTTAACATCA |
|  | R: GAAGCGCTTGTCCTGCTTTC |
| SLC8A1 | F: CCAGACACATTTGCCAGCAA |
|  | R: CTATGGAGGCGTCTGCATACTG |
| RBM20 | F: CAGAAGACATCCACGTTCCTAA  CAGAAGACATCCACGTTCCTAA  CAGAAGACATCCACGTTCCTAA |
|  | R: TTAAGCGCTCAATTCGTTTCTC |
| GPER1 | F: GAGAACGTCTTCATCAGCGT |
|  | R: AAGCTGTAGATGAGGGGGTTTA |
| IL27 | F: ACCGCTTTGCGGAATCTCA |
|  | R: AGGTCAGGGAAACATCAGGGA |
| SOCS1 | F: GAACTGCTTTTTCGCCCTTAG |
|  | R: GAAGAGGCAGTCGAAGCTC |
| GRTP1-AS1 | F: CAAGTGCCAGGCCTCACTG |
|  | R: CCAAGATGGACTCACAGCAGTCC |
| β-Actin | F: CTGGAACGGTGAAGGTGACA |
|  | R: CGGCCACATTGTGAACTTTG |
